# Analysis of spatial mobility in subjects from a Dengue endemic urban locality in Morelos State, Mexico

**DOI:** 10.1371/journal.pone.0172313

**Published:** 2017-02-22

**Authors:** Jorge Abelardo Falcón-Lezama, René Santos-Luna, Susana Román-Pérez, Ruth Aralí Martínez-Vega, Marco Arieli Herrera-Valdez, Ángel Fernando Kuri-Morales, Ben Adams, Pablo Antonio Kuri-Morales, Malaquías López-Cervantes, José Ramos-Castañeda

**Affiliations:** 1 Centro de Investigaciones sobre Enfermedades Infecciosas, Instituto Nacional de Salud Pública, Cuernavaca, Morelos, México; 2 Subdirección de Geografía Médica, Instituto Nacional de Salud Pública, Cuernavaca, Morelos, México; 3 OLFIS, Bucaramanga, Santander, Colombia; 4 Universidad de Santander, Campus Universitario, Bucaramanga, Santander, Colombia; 5 Instituto de Matemáticas, Universidad Nacional Autónoma de México, Ciudad de México, México; 6 Departamento de Computación, Instituto Tecnológico Autónomo de México, Ciudad de México, México; 7 Department of Mathematical Sciences, University of Bath, Bath, United Kingdom; 8 Subsecretaría de Prevención y Promoción de la Salud, Ciudad de México, México; 9 Unidad de Proyectos Especiales de Investigación Sociomédica, Facultad de Medicina, Universidad Nacional Autónoma de México, Ciudad de México, México; 10 Center for Tropical Diseases, University of Texas-Medical Branch, Galveston, Texas, United States of America; Georgia State University, UNITED STATES

## Abstract

**Introduction:**

Mathematical models and field data suggest that human mobility is an important driver for Dengue virus transmission. Nonetheless little is known on this matter due the lack of instruments for precise mobility quantification and study design difficulties.

**Materials and methods:**

We carried out a cohort-nested, case-control study with 126 individuals (42 cases, 42 intradomestic controls and 42 population controls) with the goal of describing human mobility patterns of recently Dengue virus-infected subjects, and comparing them with those of non-infected subjects living in an urban endemic locality. Mobility was quantified using a GPS-data logger registering waypoints at 60-second intervals for a minimum of 15 natural days.

**Results:**

Although absolute displacement was highly biased towards the intradomestic and peridomestic areas, occasional displacements exceeding a 100-Km radius from the center of the studied locality were recorded for all three study groups and individual displacements were recorded traveling across six states from central Mexico. Additionally, cases had a larger number of visits out of the municipality´s administrative limits when compared to intradomestic controls (cases: 10.4 versus intradomestic controls: 2.9, *p* = 0.0282). We were able to identify extradomestic places within and out of the locality that were independently visited by apparently non-related infected subjects, consistent with houses, working and leisure places.

**Conclusions:**

Results of this study show that human mobility in a small urban setting exceeded that considered by local health authority’s administrative limits, and was different between recently infected and non-infected subjects living in the same household. These observations provide important insights about the role that human mobility may have in Dengue virus transmission and persistence across endemic geographic areas that need to be taken into account when planning preventive and control measures. Finally, these results are a valuable reference when setting the parameters for future mathematical modeling studies.

## Introduction

Dengue fever (DF) is the most important arthropod-borne viral disease in the world. It is caused by infection with any of the four Dengue virus (DENV) serotypes. Nearly half of the human population inhabits areas with DENV transmission. In Mexico dengue incidence and severe cases have been increasing in the last decade. To date, there is no specific treatment or vaccine for DF and vector control stands as the cornerstone for DF prevention [1, 2].

DF is an important public health problem, especially in urban areas [3], where it usually presents in large outbreak. The costs of treatment and management during a DF outbreak are a serious burden for health systems, especially when there is a risk for saturation of health facilities [4].

The actors that are necessary for DENV transmission are fairly well understood. Nonetheless, some of the dynamical features about these actors still need to be elucidated in order to understand how they impact on transmission. Human mobility has been studied in relation to other infectious diseases, where its role as an important driver for disease transmission has been proven [5, 6]. Mathematical models have suggested that local scale human mobility may play a role in DENV transmission, outbreak persistence, and control efficiency [7, 8]. However, little information is available on detailed human mobility patterns in geographic areas where DF is endemic or on confirmed cases during an outbreak.

Recently, GPS-based technologies have been tested and shown to be a reliable and acceptable tool for quantifying human mobility [9, 10]. Human mobility has been described in relatively recent reports by using indirect measures [11, 12]. For these reasons, we studied the micro and macromobility of Dengue virus-infected subjects in an endemic locality. Here we present the results of a cohort-nested case-control study on a dengue endemic urban locality in Mexico.

## Materials and methods

The protocol of the project was reviewed and approved by the Comité de Etica y de Prevención de Conflictos de Interes (Institutional Review Board) CI 1046 No 1160 and Departamento de Investigación (External Review) Servicios de Salud de Morelos, Mexico DEI/CEI/0281/2012.

### Location of the study

**Fig 1:**
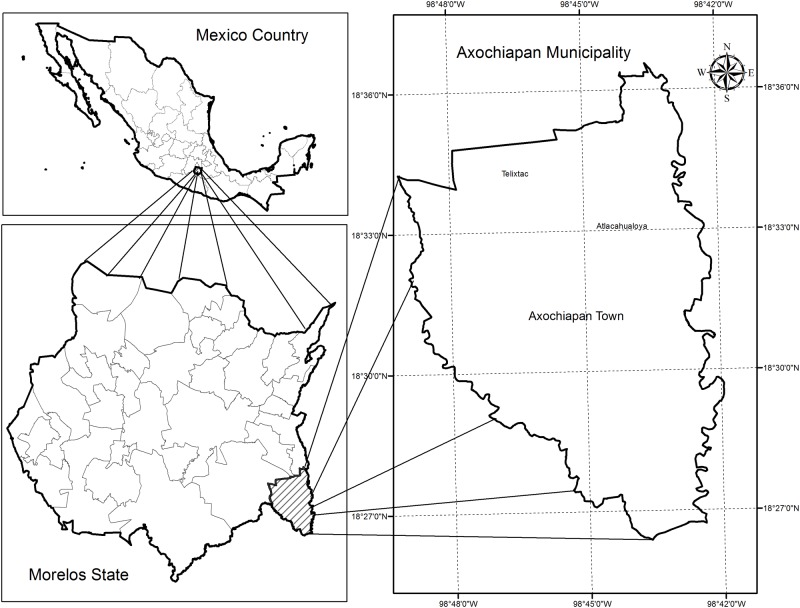
Study area. This figure shows the location of the study area (Axochiapan town) within the Axochiapan Municipality, State of Morelos and Mexico.

Axochiapan is the main locality of the Municipality of Axochiapan, in the State of Morelos México. This locality has a population of 17,505 inhabitants according to the 2010 census. Located in the position 18.50 N, -98.75 W, in the southeast corner of the State of Morelos, it shares boundaries with the State of Puebla (Fig 1).

The Municipality of Axochiapan, Morelos Mexico, is considered endemic of dengue, since it registers cases every year. Although cases can be observed at any time of the year, the endemic profile in this locality is usually seasonal with large outbreaks between May and October. The circulation of at least three DENV serotypes has been documented during the last decade; nonetheless, usually only one of them is predominant during every epidemic season and is replaced every 2–3 years causing larger outbreaks affecting all age groups. The mean incidence for the 2009–2013 period was 570 cases / 100,000 inhabitants, and the mean age for of cases was 24.

### Study type and population

**Fig 2:**
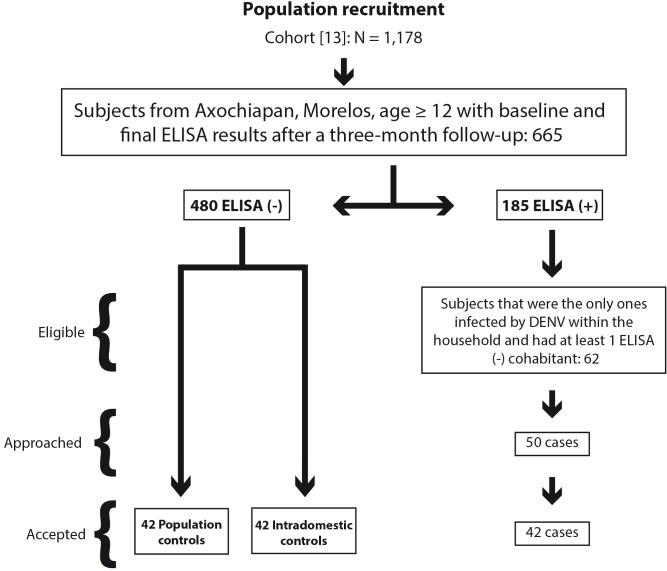
Recruitment procedure. This figure describes recruitment strategy for the participant’s selection.

Cohort-nested, case-control study. *Sample*: 126 individuals (42 cases, 42 intradomestic controls and 42 population controls) with age older than 12, and residents in Axochiapan, Morelos State, México, were selected from the cohort “Peridomestic infection as determinant for Dengue virus transmission” [13]. They were assigned into three study groups: A. *Cases* were individuals with laboratory evidence of recent, symptomatic or asymptomatic, DENV infection, and identified as the only persons infected within their households during the study. B. *Intradomestic controls*, were individuals with a negative serological result for recent DENV infection, living in the same household with a case; C. *Population controls* were individuals with a negative serological result for recent DENV infection, randomly selected from the same locality. All controls tested negative for DENV during the same period and using the same validated techniques than cases (IgM or IgG capture ELISA) as reported in the cohort study [13]. Participant´s selection was performed as follows: Cases were approached first, if accepted participation an intradomestic control was randomly assigned from the pool of subjects living in the same house that had both baseline and final negative ELISA results. For each pair of in-house participants, a randomly selected population control was then assigned (Fig 2). Given the limited number of available GPS loggers and the three-month time frame for the follow-up, the recruitment was limited to a maximum of 50 cases with their respective intradomestic and population controls.

### GPS follow-up

Between May and September, 2012, with prior signed informed consent, all participants were provided with a portable GPS (GPS Data-logger, Transystems Mod. 747 A+), programmed for recording its position at 60—second intervals (variables date, time, latitude, longitude, altitude and speed), during 24 hours a day, for a minimum period of 15 days. Participants were instructed to carry their GPS at all times whenever they left their homes and to recharge the equipment’s battery daily during their in-home resting times. This follow up was performed in cases identified during the immediate previous season, on average one year after diagnosis confirmation, in order to match the activities performed during the high transmission season, and under the assumption that their mobility patterns remained unchanged after disease, and constant through time.

### Data processing and analyses

A web—based interface was developed to import the text files from GPS equipment to a main database. Variables were homogenized, waypoints in the initial and final days of each individual GPS track (comprising incomplete days) were eliminated in order to standardize the period of time to be analyzed starting at 00:00:00 hours on the second day of follow up and ending at 23:59:59 on the last to final day of follow up. Data were converted into a feature dataset and projected from the geographic coordinate system to a Lambert coordinate system (from hexadecimal to metric units) with the purpose of performing arithmetic operations for distance calculations. Origin or routinely residence sites were identified by means of an iterative algorithm (mean center) employing waypoints from 00:00:00 to 04:59:59 hours, Monday to Friday. Routine residence coordinates were added to the database.

Distance to home variable (dhome) was calculated for each extradomestic waypoint using SQL applying the following formula:

$$dh=\sqrt[{2}]{{\left(x_{1}-x_{2}\right)}^{2}+{\left(y-y_{2}\right)}^{2}}$$

Where; x_1_ = xhome (x coordinate from home), x_2_ = xccl (x coordinate from each waypoint), y_1_ = yhome (y coordinate from home) y, y_2_ = yccl (y coordinate from each waypoint).

Waypoints within the peridomestic area (dhome < 50m) were identified.

Distance, speed, altitude and time differentials were created:

$$d=\sqrt[{2}]{{\left(Xccl_{1}-Xccl_{2}\right)}^{2}+{\left(Yccl_{1}-Yccl_{2}\right)}^{2}}$$

$$\Delta V=Speed_{i}-Speed_{i-1}$$

$$\Delta A=Altitude_{i+1}-Altitude_{i}$$

$$\Delta T=local\;date{ƒ}_{i+1}-local\;date{ƒ}_{j}$$

Where: Xccl_1_ = (x coordinate from previous waypoint), Xccl_2_ = (x coordinate from current waypoint), Yccl_1_ = (y coordinate from previous waypoint) and, Yccl_2_ = (y coordinate from current point)

Displacement and spatial permanency variables for each subject with complete data were generated.

Visit sites were defined as those areas out of the individual’s home with a 50 m radius in which each participant remained static for a period enough to allow a potential effective interaction with local vectors. These sites were identified by generating an algorithm through which visit clusters were formed using the following criteria: stops lasting 5 minutes or longer, a distance from home of 50 m or farther, distance of the current waypoint from previous waypoint < 50 m, and speed for the current waypoint of 2 km / h or less. For each cluster (visit site) a centroid was calculated.

Common visit sites for cases were identified as hexagonal cells with 50 m radius [14] which were visited by at least two different cases at a given time, and where the proportion of different visiting cases was at least two thirds of the total visiting population for that cell. Common visit sites for controls were identified as hexagonal cells with 50 m radius which were visited by at least two members from each control population and not visited by any of the cases.

The geographic universe in the study was divided in five areas (1.- Inside the house, 2.- Out of the house but in the locality, 3.- Out of the locality but in the municipality, 4.- Out of the municipality but in the state, 5.- Out of the state), limited by four buffers. A circular buffer with 50 m radius around each participant’s home limited the first area and three additional polygonal buffers were drawn according to the administrative limits for the locality, municipality and state.

ArcGIS ArcINFO 10 was used for processing and analyzing spatial data, SQL server was used to create a Geodatabase, Arc SDE 10 was used as interpreter between entre SQL and ARCGIS. Statistical analyses were performed using STATA 12.

## Results

### Sample description

Fifty randomly selected cases were asked to participate in the study from which 42 (84%) accepted participation. All approached controls agreed to participate. In total 126 individuals (42 cases, 42 intradomestic controls and 42 population controls) were recruited. Our drop-out rate was lower than 1% (1/126) since one participant (intradomestic control) did not finish the follow-up due to the loss of the assigned GPS logger. Table 1 describes the main characteristics of the subjects in each group. No statistically significant differences were observed in most of variables except in age, since cases were significantly younger than the intradomestic or population controls (Cases mean: 29.2, SD: 17.7; Intradomestic controls mean: 35.9 SD: 13.5; Population controls mean: 37.4 SD: 16.6. *p* = 0.0315).

**Table 1:** Sample characteristics.

Characteristics	Total	Cases	Intradomestic controls	Population controls	*p*
	(n = 126)	(n = 42)	(n = 42)	(n = 42)	
**Gender**					
Female n (%)	71 (56.4)	23 (54.8)	27 (64.3)	21 (50)	0.41
**Age mean (SD)**	34 (16.3)	29.2 (17.7)	35.9 (13.5)	37.4 (16.6)	0.0315
**State of birth**					
Morelos	87 (69)	27 (72.2)	28 (66.7)	32 (72.2)	0.46
**Education**					
None or basic	31 (24.6)	9 (21.4)	9 (21.4)	13 (31)	0.24
Junior high school	77 (61.1)	26 (61.9)	24 (57.1)	27 (64.3)	
High school / technician / College / University	18 (14.3)	7 (6.7)	9 (21.4)	2 (4.8)	
**Occupation**					
Student / employee / independent	72 (57.1)	30 (71.4)	21 (50)	21 (50)	0.07
Retired employee / unemployed / homemaker / other	54 (42.9)	12 (28.6)	21 (50)	21 (50)	
**Health insurance provider**					
Institutional for regular workers	7 (5.6)	2 (4.8)	3 (7.1)	2 (4.8)	0.69
Government insurance for vulnerable population	102 (81)	35 (83.3)	31 (73.8)	36 (85.7)	
None	17 (13.5)	5 (1.9)	8 (19)	4 (9.5)	

General characteristics of 126 individuals selected for participation

### Mobility description

**Fig 3:**
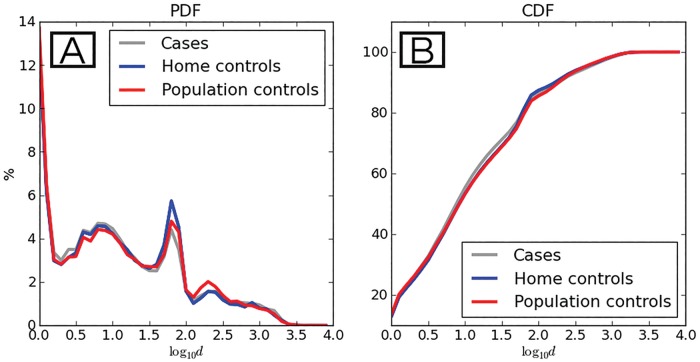
Distribution of the absolute displacement variable for each study group. (A) Probability distribution function (PDF). (B): Cumulative distribution function (CDF). Y axis: Percentage of waypoints recorded, X axis: Distance from home centroid. Distance in meters is expressed on a logarithmic scale. Line colors: gray depicts data of cases, blue depicts home (intradomestic) controls, and red depicts population controls.

Of 126 participants, 125 (99.2%) participants completed their follow up since one GPS used by an intradomestic control went missing. The final database contains 3,064,887 waypoints from these 125 participants, and all participants were followed by a mean of 15.9 continuous days. As for the number of days of follow-up for each group no differences were recorded. As expected, most of the waypoints in the population fell within the intradomestic area (< 50 m radius from home centroid). As distance from home (absolute displacement) increased, we observed a marked decrease in the proportion of waypoints. All three groups presented a small peak when the distance reached the 100 m radius. From this point the proportion of waypoints quickly decayed (Fig 3). No differences were noticed for absolute displacement among the groups.

**Fig 4:**
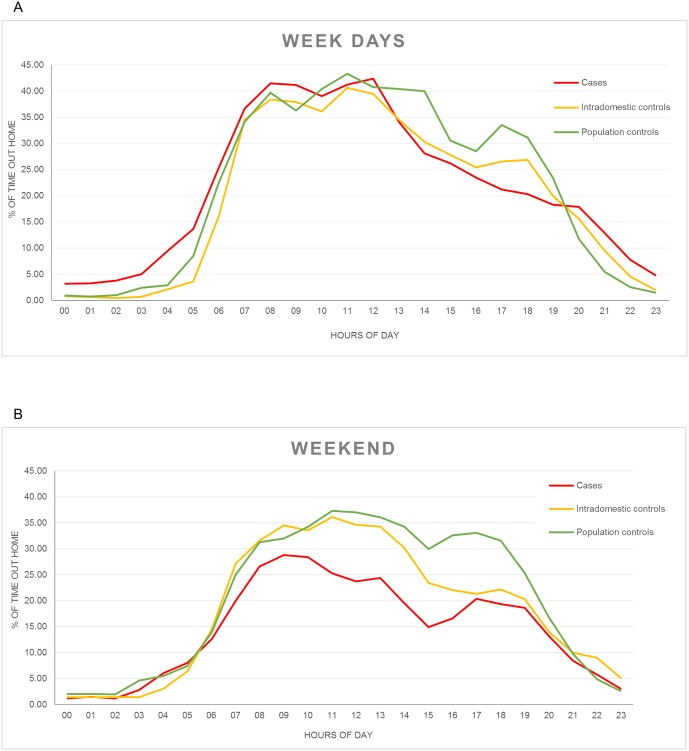
Hourly distribution of time spent out of home. (A) Proportion of time spent by the subjects out of their homes during weekdays (Monday to Friday). (B) Proportion of time spent by the subjects out of their homes during weekends (Saturday and Sunday). Y axis: Percentage of time out of the subject’s homes. X axis: hour of the day. Line colors: red depicts data of cases, yellow depicts intradomestic controls and green depicts population controls.

The hourly distribution of recorded waypoints out of the participant’s homes is shown in Fig 4. As expected, participants usually left their homes early in the morning and returned by the end of the day. Although we recorded waypoints out of the participants’ homes in every hour of the day, the period comprised between 08:00 PM and 12:00 PM registered the peak in the number of waypoints recorded out of the homes, and this number decreased steadily as the day progresses. The pattern during weekdays (Fig 4A) suggests that cases leave their homes and return to them slightly earlier than control groups. As for the weekends (Fig 4B), both control groups show a similar pattern to that observed for weekdays, nonetheless, cases seem to remain in their homes more often and return earlier.

Table 2 shows values for different mobility variables. No significant differences were recorded among groups for the following variables: mean distance from home at all times, maximum recorded distance at any given time, and mean time spent in each geographic area at any speed or at static speed. Nonetheless, when comparing the number of visits per geographic area, the cases had fewer recorded visits in the area out of the locality but in the municipality (3.1 vs 18.7, *p* = 0.0428), and more visits in the area out of the municipality (10.4 vs 2.9, *p* = 0.0282), both compared to intradomestic controls. These differences were statistically significant.

**Table 2:** Mobility description per group.

Variable	Cases	Intradomestic controls	Population controls
	(n = 42)	(n = 41)	(n = 42)
**Mean distance from home at all times in meters.**			
Mean	**2,850**	**970**	**1,076**
Median	197.1	249.3	407.6
IQR	(79.8–1,247.9)	(49.5–758.3)	(84.9–1,255.2)
**Maximum recorded distance in meters.**			
Mean	**32,226**	**19,379**	**27,078**
Median	7,362.8	4,719.4	8,466.2
IQR	(1,851.6–45,114.2)	(1,411.7–24,350.4)	(2,379.8–45,586.3)
**Mean time, in hours, spent in each geographic area during 15 days.** *Mean (range)*			
Inside their homes	329.3 (278.6–375.7)	328.4 (288.6–365.6)	353.5 (286.7–384)
Out of their homes but in the locality	46.3 (15.1–89.9)	30.6 (17.7–70.3)	55.6 (24.4–82.3)
Out of the locality but in the municipality	1.3 (0–3.8)	0.7 (0–13)	1.1 (0.17–10.8)
Out of the municipality but In State	0 (0–7.5)	0 (0–2.7)	0 (0–4.6)
Out of State	0 (0–0)	0 (0–1.4)	0 (0–3.1)
**Time, in hours, spent static (speed< 5 km/h) in each group outside the peridomestic area during 15 days.** *Mean (range)*			
Out of their homes but in the locality	56.5 (1.3–203.9)	46.8 (1.0–192.2)	58.8 (0.0–286.8)
Out of the locality but in the municipality	2.3 (0.0–15.4)	14.9 (0.0–138.3)	11.4 (0.0–93.1)
Out of the municipality but In State	3.2 (0.0–18.4)	1.1 (0.0–8.8)	3.7 (0.0–82.0)
Out of State	8.1 (0.0–205.4)	2 (0.0–18.3)	2.8 (0.0–47.7)
**# Visits per geographic area** *Mean (range)*			
All visit sites	68.9 (4–284)	75.4 (0–302)	76.4 (0–285)
Out of their homes in the locality	55.5 (3–263)	53.8 (0–299)	56.8 (0–284)
Out of the locality but in the municipality ¶	3.1 (0–30)	18.7 (0–279)	15.1 (0–157)
Out of municipality §	10.4 (0–124)	2.9 (0–26)	4.6 (0–37)
**Administrative areas visited by any member of each group and DF incidence in 2012**			
Total States visited	6	5	3
Total Municipalities visited	46	27	23
Municipalities with DF incidence >10/100,000	21	12	19
Municipalities with DF incidence >50/100,000	19	11	17
Municipalities with DF incidence >100/100,000	12	6	12

**IQR**, Interquartile range.

^**¶**^*p* = 0.0428,

^**§**^*p* = 0.0282 when comparing cases versus intradomestic controls.

Consistent with this behavior, although non-statistically significant, cases visited more States, municipalities, and regions with high dengue incidence through their follow up, in comparison to both control groups.

**Fig 5:**
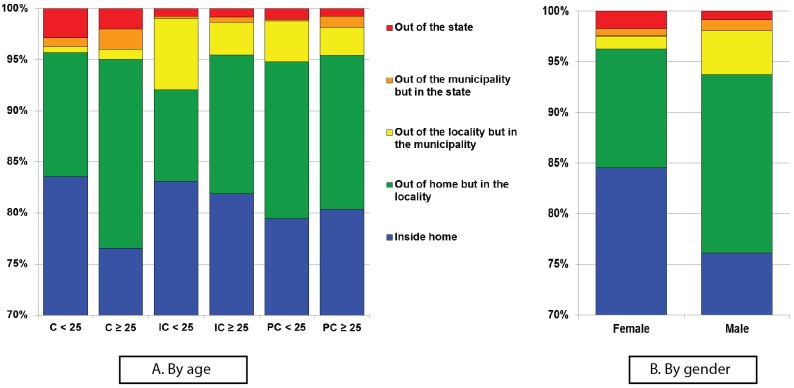
Proportion of time spent per geographic area. (A) Proportion of time spent by each group (C = cases, IC = intradomestic controls, PC = population controls) in each geographic area (out of the home, out of the home but in the locality, out of the locality but in the municipality, out of the municipality but in the state, out of the state), stratified by age (under 25 or 25 and older). (B) Proportion of time spent in each geographic area by gender.

We next examined the proportion of waypoints recorded by the comparison groups in each area stratified by age (Fig 5). There is a notorious difference in the proportion of waypoints among the cases, observing an increase of nearly 8 percentile points in the intradomestic waypoints, recording the highest frequency in cases under age 25 (Fig 5A. However the difference not statistically significant (Cases < 25: Median 90.2%, Interquartile-range 75.5–95.5; cases ≥ 25: 80% IQR 66–86.4; *p* = 0.079). We also observed a difference in the area out of the municipality but in the State, where the group of cases aged 25 and older spent the highest proportion of time (age < 25: 0% IQR 0–1%; age ≥ 25: 1.1% IQR 0–4.6; *p* = 0.0233).

There was no significant difference between cases and population controls, regardless of age. When comparing cases versus intradomestic controls, we observed a statistically significant difference in time spent in area out of the municipality but in the State (Cases: 1.1% IQR 0–4.6; IC: 0% IQR: 0–0.6; *p* = 0.009).

We found differences in mobility patterns when analyzing data by gender (Fig 5B). Women had a higher proportion of waypoints within the intradomestic area than men. These differences were statistically significant for intradomestic area (Male: 77.4% IQR: 65.8–86.9; Female: 89.4% IQR 80.5–93.5; *p* = 0.002), out of their homes but in the locality (Male: 15.5% IQR 5.3–25; Female: 7.2% IQR 4.6–16.5; *p* = 0.0063) and the area out of the locality but in the municipality (Male: 1.1% IQR: 0.1–4.9; Female: 0.1% IQR: 0–0.4; *p*<0.0001). Nonetheless, linear mean (*p* = 0.061) and maximum (*p* = 0.1468) distances were not.

#### a) Cases vs. intradomestic controls

A conditional logistic regression analysis was performed, including variables identified in the bivariate analysis as having a *p* value < 0.20, and by data mining techniques using all variables as reported previously [15]. For bivariate analysis variables were age (continuous and dichotomic [under 25 or 25 and older]) gender, occupation (intra or extradomestic), education (dichotomic), and the proportion of time spent in each geographic area. For the data mining we considered the whole data base. The final model included age (OR: 0.015 IC95% 0.0005–0.488; *p* = 0.018) and the area out of the municipality but in the State (OR 2.61 IC 95% 1.16–5.88; *p* = 0.021). We observed a protective effect in the 25 and older group, and a risk effect when the proportion of time spent in the area out of the municipality but in the State is increased.

#### b) Cases vs. population controls

A multiple logistic regression analysis was performed including variables identified with *p* value < 0.20 in the bivariate analysis and data mining techniques using all variables as reported previously [15]. For bivariate analysis, only the variables age (continuous and dichotomic [under 25 or 25 and older]), gender, occupation (intra and extradomestic), education (dichotomic), proportion of time spent in each area and linear distance were taken into consideration. For the data mining we considered the whole database.

The final model included: occupation (OR 3.02 IC 95% 1.02–8.88; *p* = 0.045), proportion of time in the area out of the municipality but in the State (1.42 IC 95% 1.02–1.98; *p* = 0.035), proportion of time in the area out of the locality but in the municipality (OR 0.74 IC 95% 0.57–0.96; *p* = 0.023), and age (OR 0.26 IC 95% 0.09–0.78, *p* = 0.017).

**Fig 6:**
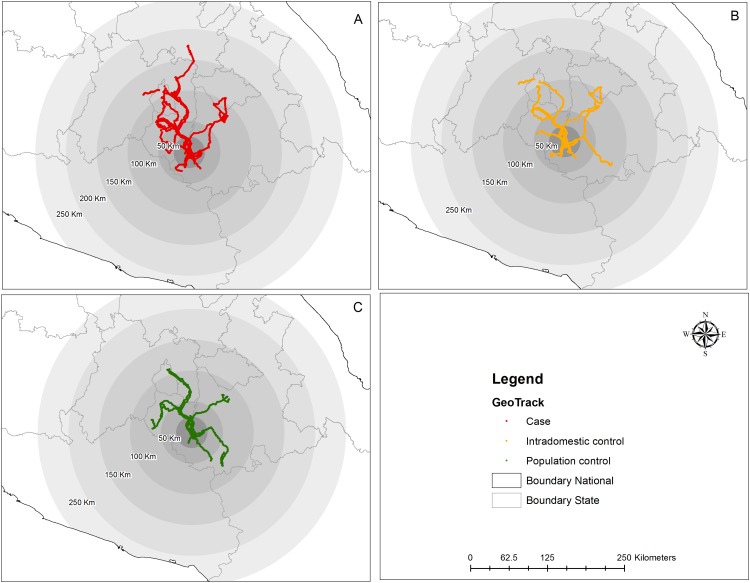
Mobility of participants across central Mexico. Figure shows the follow-up tracks for each group of participants during a 15-day period. (A) Cases, (B) Intradomestic controls, (C) Population controls. Dot colors: red shows tracks from cases, yellow shows track from intradomestic controls, green shows tracks from population controls. State boundaries and concentric distance buffers are shown for perspective.

Next we analyzed the geographic distribution of the recorded waypoints for each group, both locally and regionally (Fig 6). All three groups recorded waypoints exceeding a 100 Km radius from their homes (Fig 6A, 6B and 6C). As expected, most of recorded waypoints were located within the locality. Nonetheless, all three groups recorded waypoints exceeding the locality, municipality and state limits. These trajectories were headed mainly to the east, north and west of the locality, and were consistent with the location of the main cities in the area, including Cuernavaca (population 338,650) and Cuautla (population 154,358), the capital city and the second most important city in the state of Morelos, respectively.

**Fig 7:**
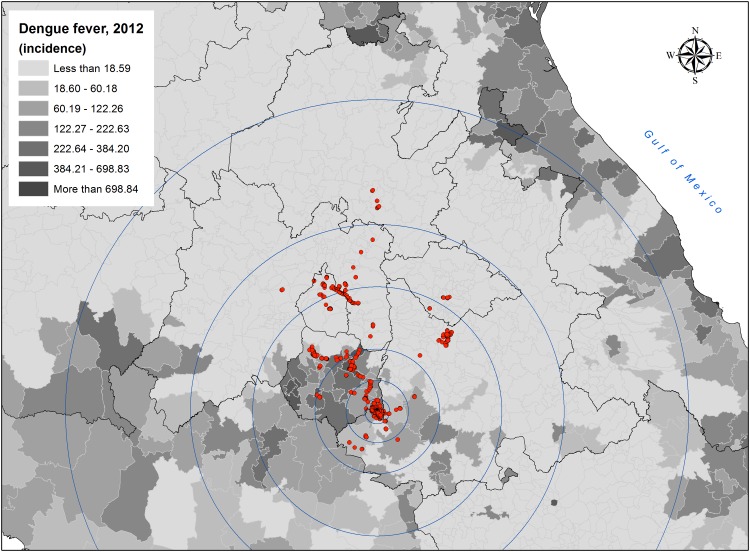
Mobility of cases across dengue endemic zones in central Mexico. The figure shows the geographic distribution of visits performed by cases across central Mexico, and the cumulative incidence of dengue at municipal level for 2012.

The geographic distribution of the visits performed by cases and DF cumulative incidence for the central Mexico region, during year 2012, is shown in Fig 7. As seen, this group performed visits to locations with and without DF transmission. The number of States, municipalities and regions with high dengue incidence is shown in Table 2.

**Fig 8:**
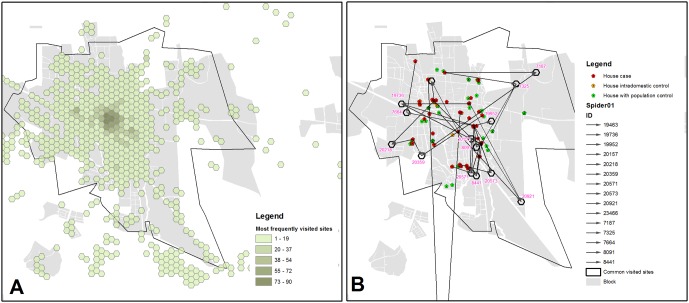
Visit sites for participants in Axochiapan town. (A) Shows the hexagonal 50-meter radius cells most frequently visited by participants regardless of the group they were allocated (cases, intradomestic controls or population controls). (B) Shows 15 hexagonal cells that were independently visited mostly by at least two cases and their respective houses as identified by GPS. Red points are the houses of cases and intradomestic controls, green points are houses inhabited by population controls, and orange points are the houses of intradomestic controls that declared living with a case but actually lived in houses different to those inhabited by a case.

Within the locality of Axochiapan the most visited areas were identified by dividing the locality in 50 m-radius hexagonal cells (Fig 8A). The most visited cells were those located in the locality’s central area, which correspond to the location of the main market, road junctions and main administrative and / or service offices. The location of the cells considered as common visit sites for cases are shown in Fig 8B. Unlike in Fig 8A, the geographical distribution of these 15 cells tends to be peripheral with respect to the locality.

Using Google Earth^™^ we identified the geographic features of each of the 15 cells that was classified as a common visit site for cases. Fourteen out of fifteen cells were geographically located within the locality of Axochiapan, Morelos, and the last one was in the central area of a neighboring small locality (Town of Tzicatlán) in the State of Puebla. As for their typology, xix out of 15 cells clearly corresponded to residential areas (including that in the neighboring State), one cell was a residential area adjacent to a local large business, four cells included small processing plants, a warehouse and a local business, three peripheral cells were crop fields and one cell was clearly a soccer field.

## Discussion

Previous works have used GPS tools for measuring exposure to infectious diseases [16–18]. In DF, recent works in the endemic area of Iquitos, Peru, have elegantly described human population mobility [19, 9]. However, few data are available for infected cases so far. Our work builds upon our knowledge of the role played by mobility in DENV transmission documenting spatial mobility of subjects from an endemic region that had, or had not been recently infected by DENV.

Our data show that the people from Axochiapan stay within their houses or surrounding areas most of the time. This is consistent with previous observations in Iquitos, where population rarely moves more than 1 km away from their homes [10], however, some individuals recorded movements to very distant locations through the relatively short follow-up period. These movements were present in all three study groups and exceeded a 100-km radius from the center of the study, covering the neighboring states of Morelos, State of Mexico, Puebla, Mexico City, Tlaxcala and Hidalgo. All of these states are located in the Mexican central plateau, which is also the best connected region in the country and therefore it is not difficult to reach those destinations by commute travel [20]. Surprisingly, spatial mobility in humans was not geographically symmetrical in our study, since no movements were recorded to state of Guerrero, which is a coastal, highly endemic area for Dengue and also a popular destination for leisure activities. As for the reason why the mobility of the individuals is biased towards central plains in Mexico and practically absent towards the southern regions, it was a surprising finding also for us, but we think that it has to do with two factors: first the economic activities in Axochiapan are mainly related to agriculture, trade and services which are strongly influenced by the needs of Mexico City and its Metropolitan area, comprised also by the States of Morelos, Puebla, México and Hidalgo. The main cities of these states were those that were visited by the cases and in a lesser extent by the controls.

Secondly, we did not perform any follow-up during summer and Christmas holidays, which In Mexico are specific periods for leisure. These activities are usually performed in places that might be different that those observed in our study, including the beaches in the southern coast. It is possible that had we performed our follow-up in vacation periods the observed mobility might have been different, and also leave us with a very interesting research question for the future.

The large size of the area covered by these few individuals from a small locality (Axochiapan has roughly 17,000 inhabitants) is of capital importance, given the fact that in Mexico, and probably in many other places, epidemiological surveillance, prevention and control activities for DF are mainly planned, supported and executed by local health authorities, who rely on the information generated by a number of systems, most of them automated [14], but that are usually restricted to their local administrative limits, namely municipality, sanitary jurisdiction or state at best. Thus when DF outbreaks overcome those limits and a wider coordination is needed, it is probably that the outbreaks are already established and the window of time for effectively applying control measures has been lost.

Our data show that the cases group had the largest difference on the time spent in the home area with strong age dependence. Older cases spent less time in their homes compared to younger cases. As far as the number of visits is concerned, subjects in the cases group, especially those aged over 25, performed many and more distant visits, than subjects in the intradomestic control group. This difference with the population control group was less marked. This scenario suggests that the population aged over 25 might play an important role in DENV persistence and dispersion perhaps working as geographic spreaders. Our group previously determined dengue incidence for this age group and proposed a dynamic model which seems to be corroborated by the results presented here [13]. Infected individuals, both symptomatic but also asymptomatic [21], may facilitate the infection of extradomestic mosquito populations in a local scale as models have suggested [8], or at a regional scale introducing or exporting the virus.

Given the fact that we performed an uninterrupted 24-hour follow up, we were capable to register the time when individuals left their homes for whatever activity they performed. To our surprise, we recorded waypoints out of the participant’s homes virtually at any hour of the day. Although the majority of records show that people in Axochiapan have a day-light pattern of activities, we recorded waypoints from cases, between 00:00 and 05:00 AM, which were consistent with participants’ declared jobs, which were related to nocturnal activities such as bakers and workers from the local stone processing plant.

The dispersion patterns described above, both in space and time, might be of importance in the results obtained in the control of DENV transmission in this and similar small localities. The usual schedule considered by local health authorities for applying preventive measures, which favors early hours for insecticide spraying and the visits by entomological control brigades, in a geographically focalized strategy might hinder the efficacy of the actions by the mere fact that people moves from their homes and remain away during the time these actions are normally applied. In our data, the hourly pattern for activity suggests that cases might leave earlier their homes during weekdays, and thus their homes might have a higher probability to remain closed by the time health authorities apply preventive or control measures.

It is important to notice the high mean age of the cases in Axochiapan, in both the participants in the study and those recorded historically in the State of Morelos, in comparison to other endemic areas from Mexico and the Americas. This is however, consistent with a previous work in the area [13], and is probably due to the fact that most (35 out of 42 members of the cases group) of the cases that we studied were asymptomatic, also, although not statistically significant, the mean age of the asymptomatic individuals was higher than that from symptomatic individuals (mean age asymptomatic: 31.54 vs mean age symptomatic: 17.43, *t* test *p* = 0.0535). Thus, suggesting a possible stronger role of asymptomatic population with age above 25 as spreaders for DENV transmission.

Previous studies have described that visiting other cases’ households is a risk factor for DENV dispersion [22]; working sites have also been suggested as possible transmission sources outside of cases’ households [23]. Models have shown that sites outside homes can play a role in DENV transmission and infection [7], and a recently published work showed positive correlations in Thailand between the *Aedes* spp. house index and specific landscape features [24]. Our findings are consistent with these data since cases coincided in houses different to their own in at least five different geographical locations. Additionally we found cases that coincided in four potential working places, and a soccer field. As far as we know, this is the first time that leisure sites have been documented as possible areas for DENV transmission. The lack of study of such sites has previously been pointed out as a weakness in the study of human mobility [25].

As for the common visit sites for controls, we identified 38 cells that were visited only by individuals from both control groups but not by cases. These cells included only one potential working site; whereas the cells commonly visited by cases included several likely workplaces.

The identification and study of the extradomestic sites where people coincide is relevant: a recent simulation model has concluded that the selection of areas for DF control out of the cases’ homes is important not only in terms of the time the subjects spend in them, but also because of the local vector:host ratio, and the other habitual destinations of people that visit the same area [8].

It is possible that much of the movement in a given society is driven by specific population needs and the possibility to fulfill them within or outside from their own locality. Axochiapan is a fairly small and well connected locality to other small cities, all of them endemic for DF. It is not unrealistic to think that some of the population needs can be readily fulfilled within the same locality whereas other cannot, compelling the population to move away in a permanent or transitory fashion. If a specific population such as that with age older than 25 becomes infected and effectively play a role as spreaders, then perhaps small and peripheral localities to larger cities might have a key importance in sustaining DENV transmission across large geographic areas. The assessment of such situations requires a critical review of existing data and the generation of specific studies that may help us to recast current models and more importantly, to completely understand urban DENV transmission [26].

We have identified some weaknesses in our study. The most relevant is our small sample size which might have hindered our capability to identify clear patterns in the mobility from this Mexican community. There is a possibility that any individual belonging to either control group might have get infected during the follow up; thus making her/him eligible to become a case, therefore disqualifying him/her for being a suitable control and consequently introducing an information bias; nonetheless, we believe that, although a possibility, this was negligible due to two reasons: first, the follow-up of 15 days was very short for this event to occur, and secondly because while recovering each GPS, we asked all individuals whether they had experienced fever or any other symptom suggesting dengue infection during the follow-up. None of the participants reported any change in their health status.

Although we understand that a more robust argument to ensure the infected/uninfected status might be performing an ELISA to each control after finishing their follow-up in order to be certain about their exposure, financial constrains made impossible this procedure.

Possible future improvements in our study are: increase the limited sample size, the inclusion of adequate representation for the population under the age of 12, which probably is a relevant group for transmission during the initial and focalized phases of a DF outbreak. Although no schools could not be identified in our study as a common site visited by cases, this observation needs to be taken cautiously since our study did not consider the follow-up of children usually studying elementary education. Thus, we cannot rule out any role of these sites in Dengue transmission at younger ages. Additionally, extending the duration of the follow up might improve the chances of successful identification of patterns whose frequencies are longer than a week, such as wage collection, bill payments, and communitarian meetings, among others. This last topic is essential; nonetheless it is limited by technical issues that might be addressed as technology for massive and continuous long-lasting follow up becomes available. Finally, the main reasons for which the participants move were not deeply explored in our study, thus we cannot be certain whether the recorded movements indeed depend on non-satisfied needs or on leisure activities. We can only assume that at least those movements performed during the mornings and afternoons between Monday and Friday correspond to real needs such as employment, education, and supply acquisition, and those performed during weekends are related to leisure.

Some causes for loss of GPS information in field studies have been recently described [27]. Although some of these causes might be present in our study, we believe they did not represent significant sources of bias or information loss, since we took some specific measures. For example, people were prevented of accidentally turning the GPS off by strapping a tape in the controls. In order to diminish the probability that the participants could forget their GPS units at home we performed a weekly phone call reminding them the importance of the usage attachment according the protocol during each individual follow-up. Barriers to signal were not important in the studied area since it is located in a plateau with few elevations, and buildings taller than 3-stories are practically absent. Finally, the GPS equipment used in the study had battery autonomy of up to 32 straight hours and enough memory for recording up to five times the mean number of waypoints programmed to collect in each subject.

Based on our own data and that from recently published works we conclude that GPS-based technology is a solid tool for the study of detailed human mobility in DENV transmission or other infectious diseases, which can and must be adopted in public health and epidemiology as a basic instrument. The important geographic dispersion in our results demonstrates the necessity for studying the potential role that human mobility has in DENV transmission and outbreak duration and also a strong argument to study and clarify the role that asymptomatic cases might have in Dengue virus dispersion. Furthermore our data strongly suggest that the size of the areas considered for prevention and control of DF outbreaks needs to be revised and that it is necessary to integrate this knowledge into the planning of preventive and control measures, which usually are prone to using basic shapes such as circles or squares as geographic references in order to define limits, ranges, trajectories and points of origin. It is clear that human populations move normally across geographical areas and not only during holidays or vacations. According to our data, the magnitude of these displacements is larger than that considered as an administrative responsibility for local health services providers. This is relevant for DENV transmission if a large fraction of that mobile commuting population is also asymptomatic but viremic, facilitating with their movements the exposure of local uninfected mosquito populations with the virus, which might result in an increased geographical dispersion and persistence of the outbreaks due a continuous process of spreading and reintroduction of the virus to susceptible populations.

Finally, we believe that the data here reported should be valuable for parameterization of mathematical models exploring specific issues in dengue epidemiology such as geographical dispersion of human activities, contact rate among humans in intermediate spots, optimal range for vector control coverage, optimal target places for health promotion activities, impact of coordinated regional collaboration, and transmission dynamics among satellite and large cities. All essential topics that are still to be understood and weighed as drivers in the transmission of this and other mosquito-transmitted diseases.
